# Synthesis and DFT investigation of new bismuth-containing MAX phases

**DOI:** 10.1038/srep18829

**Published:** 2016-01-07

**Authors:** Denis Horlait, Simon C. Middleburgh, Alexander Chroneos, William E. Lee

**Affiliations:** 1 Department of Materials and Centre for Nuclear Engineering, Imperial College London, London SW7 2BP, United Kingdom; 2 IME, Australian Nuclear Science and Technology Organisation, Lucas Heights, New South Wales, Australia; 3 Westinghouse Electric Sweden, SE-721 63 Västerås, Sweden; 4 Faculty of Engineering, Environment and Computing, Coventry University, Priory Street, Coventry CV1 5FB, United Kingdom

## Abstract

The M_n + 1_AX_n_ phases (M = early transition metal; A = group A element and X = C and N) are materials exhibiting many important metallic and ceramic properties. In the present study powder processing experiments and density functional theory calculations are employed in parallel to examine formation of Zr_2_(Al_1−x_Bi_x_)C (0 ≤ x ≤ 1). Here we show that Zr_2_(Al_1−x_Bi_x_)C, and particularly with x ≈ 0.58, can be formed from powders even though the end members Zr_2_BiC and Zr_2_AlC seemingly cannot. This represents a significant extension of the MAX phase family, as this is the first report of a bismuth-based MAX phase.

The MAX phases are a family of ternary carbides and nitrides. To be designated as a MAX phase, a compound should crystallize with the hexagonal *P*6_3_/*mmc* structure in the general formula M_n + 1_AX_n_ with n being an integer, M being an early transition metal, A being a group 13–16 element and X being C and/or N^1^. More than half of known MAX phases were first synthesized in the 1960s by Nowotny *et al.*^2^ They were little studied for more than two decades until Barsoum and El-Raghy reported in 1996 the remarkable properties of Ti_3_SiC_2_^3^ and further demonstrated these properties were shared by the other MAX phases^4^,^5^. Indeed, due to their structure consisting of the stacking of n “ceramic” layer(s) of MX interleaved by an A “metallic” plane, MAX phases are characterized by a combination of both ceramic and metallic behavior. Like most metals and alloys, they possess high thermal shock resistance, have a good machinability, and high thermal and electrical conductivities while like most ceramics they have high decomposition or melting temperature and high elastic stiffness^1^. Furthermore, MAX phases are also of interest as precursors for MXenes (M_n + 1_X_n_ two-dimensional nanosheets with properties analogues to graphene) currently only synthesizable by selective chemical etching of mostly Al-based MAX phases^6^,^7^.

From 1996 to 2004, intensive research expanded the MAX phase family to more than 60 compositions that included 9 different “M” elements, 12 different “A” elements and n values of 1, 2, and 3^1^,^8^,^9^, Since then, new discoveries reported have mainly focused on MAX phases with higher n values and on solid solutions^9^,^10^,^11^, *i.e.* partial substitution of the M, A or X elements, hence broadening to a quasi-infinite number of possible combinations. In 2014, Naguib *et al.*^10^ listed 68 quaternary MAX phases compositions already reported. Quaternary MAX phases also offer the possibility of including new elements that do not form alone a bulk ternary MAX phase, such as Mn recently incorporated in (Cr_1−x_Mn_x_)_2_GaC (0 ≤ x ≤ 0.3) by Mockute *et al.*^12^

The crystal structure of the M_2_AX phases (or *211* MAX phases) is given in Fig. 1. For the M_2_AX phase the structure can be described as a highly symmetric hexagonal unit cell that contains atomic layers of the constituent elements stacked along the *c* direction. Two M layers enclose an X layer of atoms forming an M_2_X slab with a local fcc-type stacking sequence. Atomic layers of A separate the M_2_X slabs. Local stacking around the A layer has an hcp pattern and thus the A layer forms a mirror plane in the crystal. The crystal structure can be defined by the *a* and *c* lattice vectors and the interplanar separation between the M and X atomic layers (d_MX_).

A potentially significant application of MAX phases is their use by the nuclear industry. Ion and neutron irradiations studies performed since 2009^13^,^14^, determined the good resistance (low swelling, limited decomposition or amorphisation) of MAX phase carbides and notably of Ti_3_SiC_2_. These properties make MAX phases candidate materials *e.g.* in the capacity of protective coatings for zirconium alloy nuclear fuel cladding. As tragically reminded by the recent events of Fukushima, in the case of a Loss-of-Cooling Accident (LOCA) and the impossibility to temper the reactor temperature, Zr clads can react over about 1200 °C with residual steam to catalytically produce H_2_, eventually leading to release of nuclear fuel in the reactor and to dramatic explosions. A way to avoid or at least postpone this catalytic reaction is via the protection of the nuclear fuel clad against high-temperature oxidation. Among the MAX phases, Zr_2_AlC would be advantageous as 1) Zr has a very high neutron transparency and is the main constituent of the substrate clad, 2) Al by its presence on the A metallic planes is likely to readily diffuse out to form an alumina protective layer appropriate for high temperature oxidation protection analogously to what is achieved for Ti_2_AlC*1* and 3) C, as opposed to ^14^N does not lead to problematic activation products. Nevertheless, to our knowledge, there are no reports on the synthesis and thereby on the actual existence of Zr_2_AlC. It can be argued that other non-MAX phase ternary layered carbides such as Zr_2_Al_3_C_4_ could be suitable^15^, however the reported oxidation resistance of these carbides is insufficient^16^.

One possible way then to obtain a material having similar properties as those anticipated for Zr_2_AlC is to partially substitute one of the constituting elements by another one stabilizing the structure. As the number of possibilities to test is experimentally constraining, an informed pre-selection of compositions to investigate is necessary. In this sense and in respect to our targeted nuclear application, elements with high neutron cross-section (i.e. Hf, Ta, Cd and In notably) have to be avoided. Then, density functional theory (DFT) calculations can provide important insights into the material properties and phase stability of the candidate M_n + 1_AX_n_ phases that can be complementary to experimental studies^17^,^18^,^19^,^20^,^21^. Notably, for some of the M_n + 1_AX_n_ phases there is little empirical information as experiments are hindered by the difficulty of producing single phase samples^22^,^23^. Considering phase stability, previous DFT studies^24^,^25^,^26^ have correctly predicted the stability (or not) of M_n + 1_AX_n_ phases with respect to their competing phases.

In the present study DFT is employed to investigate the phase stability and structure of Zr_2_(Al_1−x_Bi_x_)C (0 ≤ x ≤ 1) while attempts were performed in parallel to form Zr_2_(Al_1−x_Bi_x_)C by powder processing.

## Results and Discussion

### Syntheses and characterizations

Heat treatments at 1300 °C and 1450 °C were initially chosen considering the conditions reported for the synthesis of other Zr_2_AC MAX phase^4^,^27^,^28^,^29^,^30^ to try producing Zr_2_BiC, Zr_2_(Al_0.50_Bi_0.50_)C and Zr_2_AlC. On the basis of initial results, a 1150 °C treatment was also tried. Phase determinations from X-ray diffraction (XRD) of all attempted syntheses are summarized in Table 1. Neither Zr_2_AlC nor Zr_2_BiC were obtained, whatever the temperature of reaction. This is consistent with the absence of reports of Zr_2_AlC and Zr_2_BiC syntheses, although 5 other Zr_2_AC MAX phases can be formed in similar experimental conditions (A = S, Sn, In, Tl or Pb)^4^,^27^,^28^,^29^,^30^,^31^. This also confirms that Zr_2_AlC and Zr_2_BiC are not (meta-)stable and/or are less stable than the combination of ZrC + ZrAl_2_ or ZrAl_3_ and ZrC + Bi + one unknown phase respectively obtained instead (Table 1).

More importantly, XRD determined that the attempt to synthesize Zr_2_(Al_0.50_Bi_0.50_)C at 1300 °C was partially successful as a MAX phase was formed along with ZrC and ZrAl_2_ (Fig. 2). During Energy Dispersive X-ray spectrometry (EDX) characterization, point analysis of over 10 independent MAX phase grains gave elemental ratio of (Al + Bi)/(Zr + Al + Bi) of 0.33(3), hence agreeing perfectly with a *211* MAX phase, and a Bi/(Al + Bi) ratio of 0.58, with a dispersion of only±0.02, indicating the formation of Zr_2_(Al_0.42_Bi_0.58_)C. Note that the deviation from the targeted stoichiometry 1:1 Al:Bi is in agreement with the presence of ZrAl_2_ as a secondary phase, accommodating excess Al. Furthermore, Spark Plasma Sintering (SPS) was realized on the 1300 °C Zr_2_(Al_0.50_Bi_0.50_)C sample at the same temperature at 35 MPa for 10 minutes. Unexpectedly, the MAX phase content in the resulting densified ceramic sample dropped almost to zero with ZrC and ZrAl_2_ XRD patterns rising concomitantly. As it has never been reported that such pressure can decompose a MAX phase, it is more likely the decomposition temperature of the Zr_2_(Al_0.42_Bi_0.58_)C MAX phase lies somewhere around 1300 °C and is more advanced in SPS due to a temperature overshoot at the end of the heating ramp and/or because the vacuum helps Bi evaporating out of the system during the decomposition process. This concurs with Barsoum *et al.*^4^ and El-Raghy *et al.*^28^ works who found, respectively for Zr_2_SnC and Zr_2_PbC, that the decomposition temperature of such MAX phase is around 1300 °C.

From these initial findings, a lower temperature of synthesis was chosen, 1150 °C, and was employed to attempt to synthesize the same Zr_2_(Al_1−x_Bi_x_)C compounds with x = 0, 0.50 and 1. As it was anticipated the Zr_2_(Al_0.42_Bi_0.58_)C compound obtained at 1300 °C could be the result of a particular structural stabilization process, a powder mix targeting Zr_2_(Al_0.41_Bi_0.59_)C was also prepared and fired at 1150 °C (x value of 0.59 was selected instead of 0.58 to comply with the ratio 19/32 ≈ 0.59 which was the closest ratio achievable in the DFT calculations). Here again Zr_2_BiC and Zr_2_AlC syntheses were unsuccessful, whilst for mixed compositions a single MAX phase was produced as the main phase as reported in Fig. 2. Figure 2 reveals that the relative ZrC content decreases with decreasing synthesis temperature as well as when targeting Zr_2_(Al_0.41_Bi_0.59_)C instead of Zr_2_(Al_0.50_Bi_0.50_)C. It is interesting to note that for all three syntheses that lead to the formation of a MAX phase, EDX characterization always gave an average Bi/(Al + Bi) ratio of 0.58 (±0.02 to 0.04 scatter). This is indirectly confirmed by comparing the unit cell parameters determined by XRD data refinement as the x = 0.50 and x = 0.59 syntheses at 1150 °C have very close unit cell parameters and corresponding volumes (*a* = 3.335(5) Å, *c* = 14.51(2) Å, V = 139.8(4) Å^3^ and *a* = 3.344(5) Å, *c* = 14.51(2) Å, V = 140.6(5) Å^3^, respectively).

Figure 3 is a representative SEM image of Zr_2_(Al_0.42_Bi_0.58_)C grains (composition checked by EDX on this precise location). The powders consist of 2 to 10 microns agglomerates of stacked rounded platelets. Interestingly, this microstructure and grain size does not differ as a function of targeted composition (Zr_2_(Al_0.41_Bi_0.59_)C vs. Zr_2_(Al_0.50_Bi_0.50_)C) or of temperature (1150 vs. 1300 °C). SEM imaging of large areas (≈10.000 μm^2^) of synthesized powders reveals that such agglomerates represent a vast majority of the volume (around 90 vol.% for 1150 °C Zr_2_(Al_0.41_Bi_0.59_)C), which suggests the samples consist mainly of Zr_2_(Al_0.42_Bi_0.58_)C. Again the synthesis temperature was found to have little effect on sample phase composition and the only actual difference, revealed by XRD (Fig. 2) and SEM/EDX between 1150 and 1300 °C is the nature and relative ratios of the detected secondary phases (ZrC, Zr_5_Al_4_, ZrAl_2_).

### Density functional theory calculations

Nine stoichiometries were considered for DFT calculations: x = Bi/(Al + Bi) = 0, 0.25, 0.34, 0.41, 0.50, 0.59, 0.66, 0.75 and 1. The formation enthalpy of all nine stoichiometries was calculated from the constituent elements, as described in the methodology. The formation enthalpy of the Zr_2_AlC compound was calculated to be −0.40eV and the formation enthalpy of the Zr_2_BiC was calculated to be −4.17eV. Both are negative indicating that they are more stable compared to their constituent compounds (Zr + C + Al or Bi). The formation enthalpies for all stoichiometries are reported in Fig. 4. For Zr_2_(Al_1−x_Bi_x_)C solid solutions, the formation enthalpies are close but however all slightly inferior to the values expected considering a proportionality between Zr_2_BiC and Zr_2_AlC end-members values.

The mixing enthalpies of the compounds (mixed from a stoichiometric combination of Zr_2_AlC and Zr_2_BiC) are now discussed. As can be ascertained from Fig. 5, all mixing enthalpies of the considered stoichiometries are negative. Therefore DFT calculation predicts that quaternary Zr_2_(Al_1−x_Bi_x_)C will be formed preferably rather than a mixture of Zr_2_AlC and Zr_2_BiC, at least for the considered x range (0.25 ≤ x ≤ 0.75). This is in agreement with our experimental results finding formation of Zr_2_(Al_0.42_Bi_0.58_)C over Zr_2_AlC + Zr_2_BiC.

Another relevant piece of information from DFT calculations is that amongst each series of ten Zr_2_(Al_1−x_Bi_x_)C simulation boxes, the Al and Bi starting repartitions are random but different. In spite of this, there is no outlier and the dispersion of obtained results is low (this is evidenced by the low dispersion obtained in Fig. 5, ~ ± 0.01 eV per unit cell). This implies Al and Bi repartition do not seem to have any importance on the structure stability and therefore suggests that the relative higher stability of Zr_2_(Al_1−x_Bi_x_)C with 0.25 ≤ x ≤ 0.75 over the other compositions is presumably not experimentally achieved through an ordering of the two A elements.

The mixing enthalpy values are close one to another and Fig. 5 therefore does not help in understanding why the x = 0.58 composition appears to form experimentally over other stoichiometries. Further analysis of the data produced by DFT calculations may however give some hints on the origin of the enhanced stability of Zr_2_(Al_0.42_Bi_0.58_)C and conversely explain the non-stability of the end-members. The following paragraphs and Figures therefore aim to present the evolution of some relevant features observed in the simulations.

**Figure 1:**
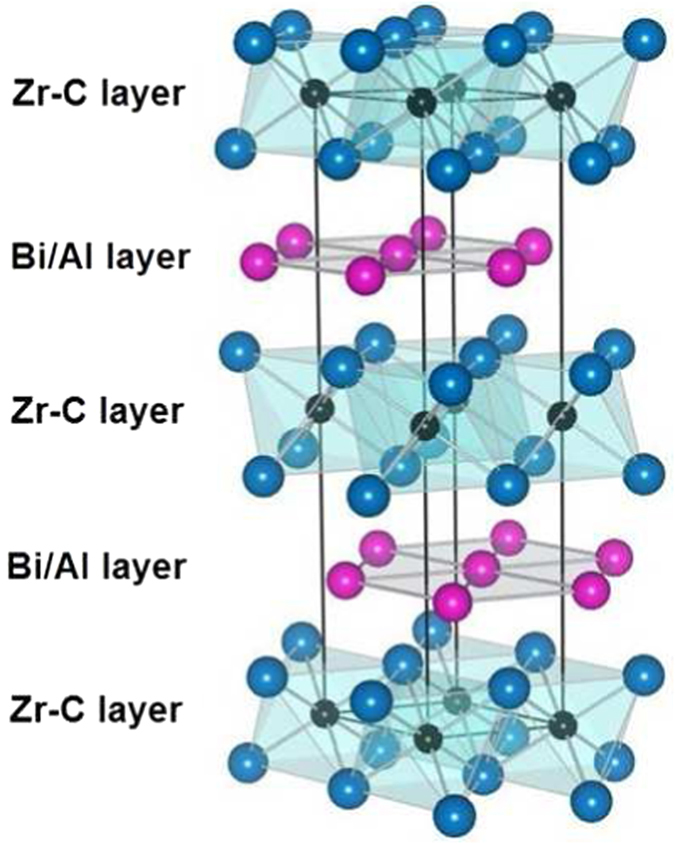
Representation of the *P*6_3_/*mmc* crystalline structure of the *211* MAX phases. Blue atoms: Zr; black atoms: C and pink atoms: Bi or Al. The unit cell is delimited by the plain black lines.

**Figure 2:**
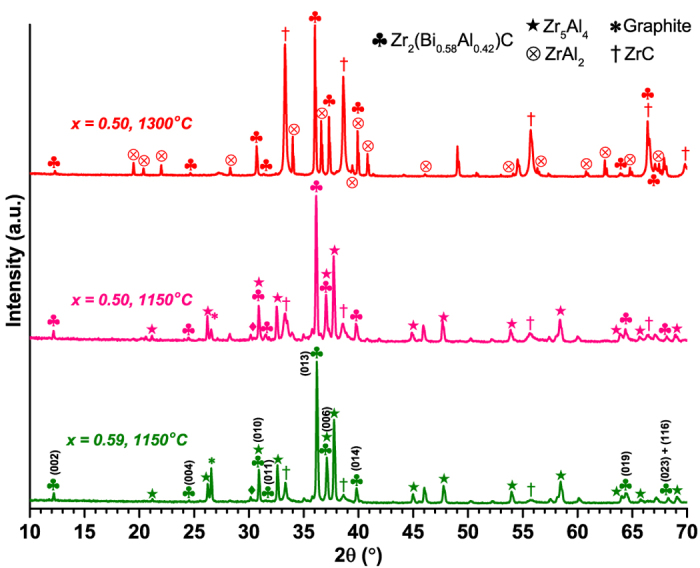
X-ray diffractograms of ZrH_2_ + Bi + Al + C powder mixes reacted for 10 h at 1300 or 1150 °C for Bi/(Al + Bi) ratios of 0.50 and 0.59.

**Figure 3:**
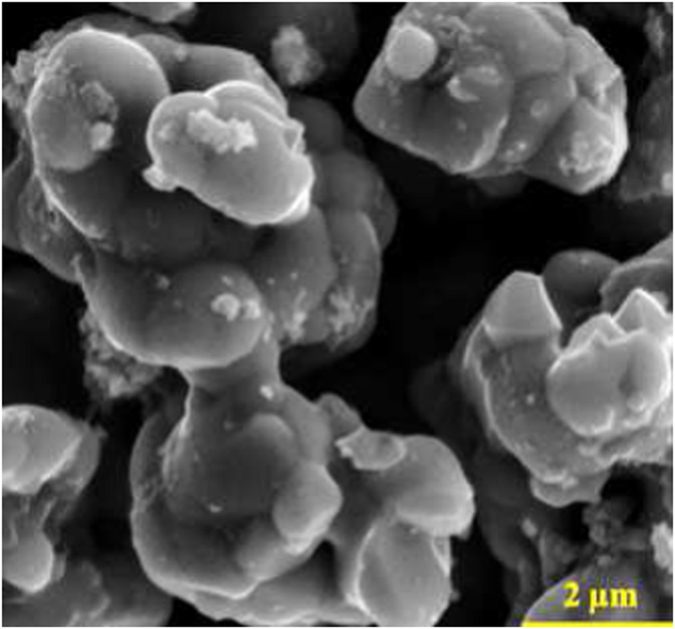
SEM image (secondary electron imaging mode) of Zr_2_(Al_0.42_Bi_0.58_)C powder (Zr_2_(Al_0.41_Bi_0.59_)C synthesis attempt at 1150 °C).

Figure 6a presents the evolution of unit cell parameters determined by the *ab initio* calculations. The *a* lattice parameter monotonically increases as a function of x (from Zr_2_AlC to Zr_2_BiC, *a* increases by 4.5%). In other words, the C-C (first neighbors) distances and therefore the (0001) plane of the unit cell (Fig. 1) are proportionally expanding with Bi incorporation. The *c* parameter variation is quite different as the maximum *c* lattice parameter is obtained for x = 0.59, i.e. the experimentally most stable composition (Zr_2_(Al_0.42_Bi_0.58_)C), then unexpectedly drops between x = 0.75 and 1 for a reason that is explained later in this paper. The difference between the maximum and minimum *c* values (at x = 0.59 and x = 1 respectively) is of 1.0%, therefore 4.5 times less than *a* maximum variation, meaning that changes in the A element size and nature mainly impact the *a* lattice parameter. Figure 6b shows that the unit cell volume increases with Bi incorporation, as expected by the larger size of Bi compare to Al. The cell volume increases proportionally to x and follows rather well Vegard’s law (i.e. the proportional variation between the two end-members).

In Fig. 7 the evolution of first neighbors Zr-C distances are reported. An important decrease is first seen between Zr_2_AlC and Zr_2_(Al_0.75_Bi_0.25_)C. It is expected by the incorporation of larger Bi atoms (Bi metallic radius is 1.70 Å, Al is 1.429 Å) which expands the A layer in the structure therefore compressing the Zr_2_C blocks. For the quaternary compositions it is seen that the Zr-C distances are increasing by 0.8% between x = 0.25 and x = 0.75. Therefore the increase in incorporation rate of larger Bi into the structure is found by DFT calculations to be accompanied by a relaxation of the Zr-C bonds. At the same time the *c* lattice parameter is calculated to be little impacted by Al substitution by Bi, while *a* is greatly increased (+0.26% and + 2.0% in the same interval for *a* and *c*, respectively). It is thus concluded that for the treated quaternary compositions, the increase in incorporation rate of Bi is accommodated through a compression of the Zr-C layers along the *c*-axis itself mostly relaxed by an expansion along the (0001) plane.

Figure 7 also reveals the Zr-C distances for Zr_2_BiC are far greater than those of the quaternaries (+4%). In view of the lattice dimensions evolution reported in Fig. 6, this result was quite unexpected. Such steep variation of property between Zr_2_(Al_0.25_Bi_0.75_)C and Zr_2_BiC was also found when drawing up and comparing the density of states (DOS) (Fig. 8). While the DOS of the quaternary compositions are similar to each other and are typical of those of other *211* MAX phases ^1^,^32^, that of Zr_2_BiC has little or no electron density at the Fermi level, creating a pseudogap (Fig. 8). This implies that the bonding nature between Bi and Zr is abnormally more covalent and less metallic than in most of the other MAX phases, including the quaternary systems for which somehow the replacement of ¼ of the Bi by Al prevents most if not all of such high covalence of the Zr-A bond. As a consequence of the enhanced Zr-Bi covalence in Zr_2_BiC, the Zr-Bi bond shrinks and so does the structure along the *c*-axis, retrospectively explaining the drop in *c* observed in Fig. 6a between x = 0.75 and 1. A similar set of features (DOS and *c* lattice parameter) has been previously encountered and reported by Barsoum^32^ for Ti_2_SC and given the relatively low *c*-parameter of Zr_2_SC, the latter should be part of this small group of outliers. Even if this atypical most-stable state found by the DFT calculations does not explain why Zr_2_BiC appears experimentally non-synthesizable contrary to Zr_2_(Al_0.42_Bi_0.58_)C – Ti_2_SC is a living proof that a peculiar bonding structure can be (meta-)stable – it however presumably proves that the better stability of the quaternary compositions compared to Zr_2_BiC arises from Al presence precluding Bi to lose its metallic characteristic.

### Discussion and summary

At this point the question is “Why can we produce a quaternary Zr_2_(Al_1−x_Bi_x_)C while we seemingly cannot produce any of the two ternary end members?” And following this, we can also wonder “What is so special about Zr_2_(Al_0.42_Bi_0.58_)C composition so that this later forms over other stoichiometries?” With the results available, this second question can hardly be answered. Aside from the fact that DFT calculations along a compositional range are rarely finding exactly the same threshold tipping values as the ones found in real experiments, none of the DFT-derived results clearly highlight a particular behavior for a certain x value in the 0.25 ≤ x ≤ 0.75 range. The only notable point is that the lowly varying *c* lattice parameter is maximum for Zr_2_(Al_0.41_Bi_0.59_)C suggesting the experimental selection of the x = 0.58 stoichiometry may be driven by steric considerations.

About the apparent non-stability of Zr_2_AlC and Zr_2_BiC while Zr_2_(Al_0.41_Bi_0.59_)C is stable, this is actually a more wide open question, since despite many efforts ^1^,^33^, we still lack a comprehensive picture of what is driving ternary and *a fortiori* quaternary MAX phases’ stability. That is, advanced DFT calculations can predict fairly well whether a MAX phase is stable or not and whether it will form over competing phases or not if the formation enthalpies of these competing phases are also calculated^1^,^23^,^24^,^25^,^33^. DFT and other experimental data comparison also pointed out *c*/*a* ratios, valence electron concentration^1^ and shear modulus/bulk modulus ratio^33^ are likely to be key parameters regarding stability but however at the current stage, all these DFT related works have not been able to elicit a simple and universal predicting rule from constituting elements characteristics such as *e.g.* ionic radius or *c*/*a* ratios. Once again a presumable main part of the problem should be that a MAX phase existence does not only depend on its own structural stability but also on that of all possible competing phases^24^,^25^.

**Figure 4:**
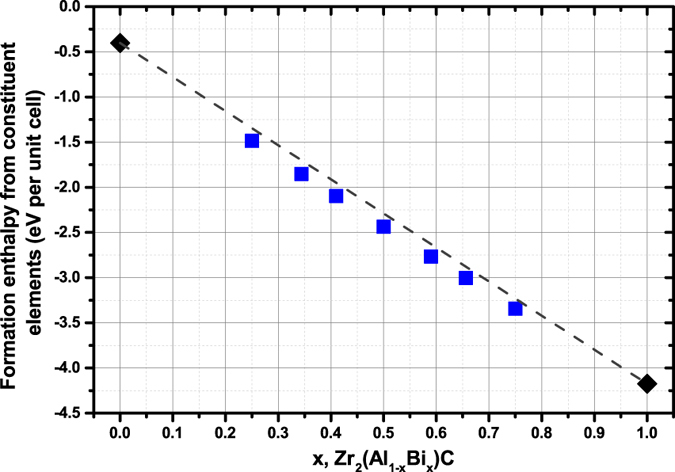
Variation in formation enthalpy of Zr_2_(Al_1−x_Bi_x_)C per unit as a function of Bi content. The gray dashed line represents the proportional variation between the two end-members.

**Figure 5:**
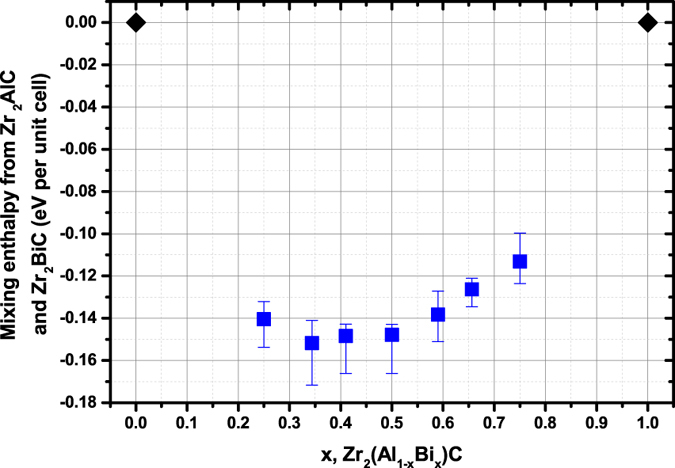
Variation in mixing enthalpy of Zr_2_(Al_1−x_Bi_x_)C per unit cell as a function of Bi content. Each error bar give the minimum and maximum values determined out of 10 independent simulation box while each square dot gives the average value.

**Figure 6:**
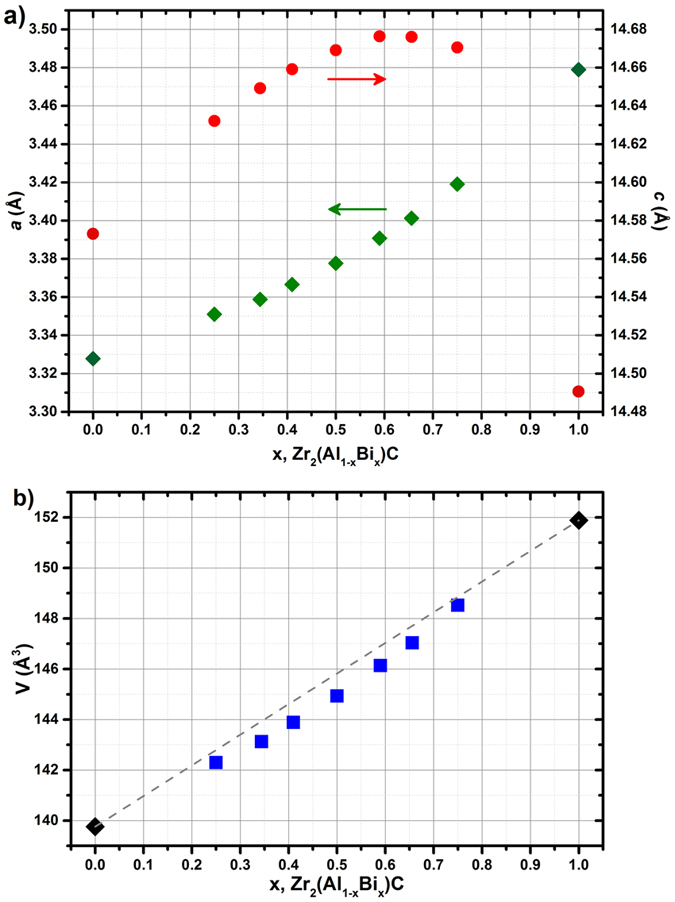
Variation of (a) the *a* and *c* lattice parameters and of (b) the corresponding volume with the Bi content in Zr_2_(Al_1−x_Bi_x_)C. The gray dashed line in (**b**) represents the proportional variation of volume between the two end-members.

**Figure 7:**
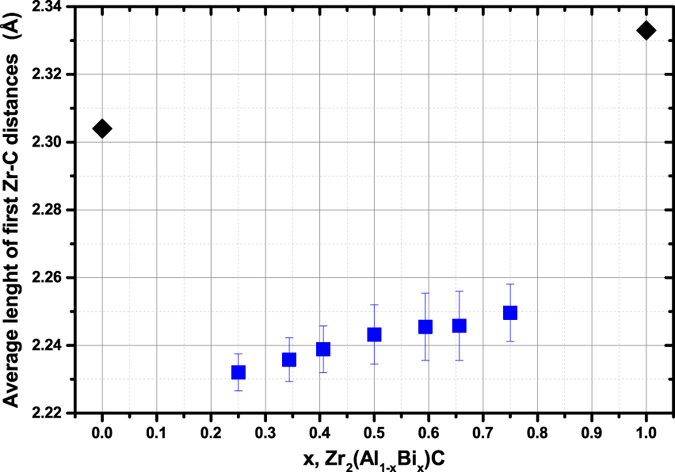
Variation of the average first Zr-C distance with the Bi content in Zr_2_(Al_1−x_Bi_x_)C determined from the DFT calculations. The error bars correspond to the standard deviation of all measured distances.

**Figure 8:**
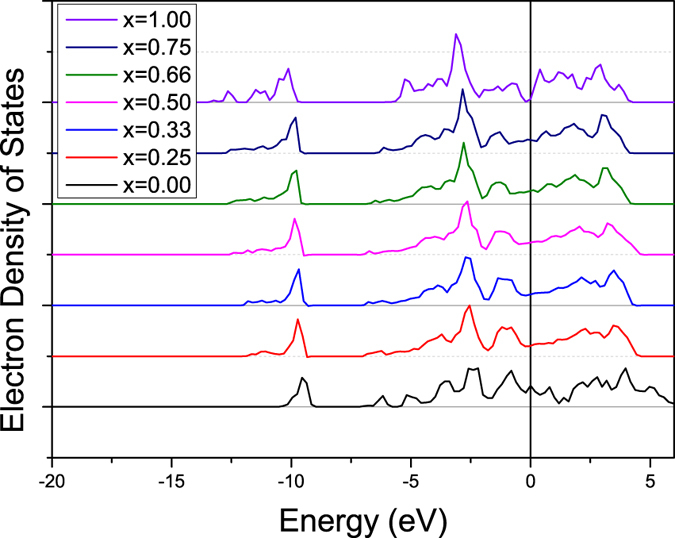
Density of states of Zr_2_(Al_1−x_Bi_x_)C. The 0 eV value corresponds to the Fermi level.

In the present case, the simplest possible answer to the first question obviously is that Zr_2_AlC and Zr_2_BiC are structurally less stable than the combination of phases experimentally obtained (Table 1). Nonetheless it is also likely that the experimental formation of a MAX phase can be precluded because of a disadvantageous combination of onset temperatures of formation (T_f_) and of decomposition (T_d_) and potentially of the associated kinetics (k_f_ and k_d_) of the two reactions. As discussed before in this paper, T_f_ and T_d_ are often close for Zr_2_AC MAX phases^4^,^28^. It is then possible we do not obtain either Zr_2_AlC or Zr_2_BiC because T_d_ 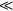 T_f_ or because T_d_ ≈ T_f_ but for the tested temperatures k_d_ 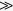 k_f_. Whatever the reason for the experimental absence of Zr_2_AlC and Zr_2_BiC although nothing from DFT works directly suggests a non-stability^34^,^35^,^36^,^37^, the quaternary Zr_2_(Al_0.42_Bi_0.58_)C somehow produces an enhanced structural stability, which results in rendering the formation of the MAX phase more energetically favorable than competing phases and/or in favorably changing the T and k above-defined values, so that formation takes over decomposition. As it can be simply seen with a periodic table and the list of so far synthesized MAX phases, expectations about Zr_2_AlC stability (at least compared to ZrC + Zr_y_Al_z_) have to be low since none of the Al neighbors (Si, Ga and Ge) forms a Zr_2_AC MAX phase^4^,^27^,^28^,^29^,^30^,^31^. Conversely some of Bi neighbors forms a Zr_2_AC MAX phase (A = Pb, Sn). That and the lower formation enthalpy of Zr_2_BiC compared to Zr_2_AlC (Fig. 4) strongly suggests Bi presence is stabilizing the MAX structure. However, too much Bi (or too little Al) was predicted by DFT to render Bi much more covalent in Zr_2_BiC than usually seen in MAX phases. This difference of Bi behavior in Zr_2_BiC compared to Zr_2_(Al_1−x_Bi_x_)C (0.25 ≤ x ≤ 0.75) is therefore likely to be directly or indirectly responsible for the apparent non-stability of Zr_2_BiC although Zr_2_(Al_0.42_Bi_0.58_)C is experimentally stable.

In summary, the present study has considered the synthesis of Zr_2_(Al_1−x_Bi_x_)C MAX phases. It is determined here that Zr_2_(Al_1−x_Bi_x_)C and especially Zr_2_(Al_0.42_Bi_0.58_)C forms in contrast to Zr_2_BiC and Zr_2_AlC that seemingly do not. DFT supports the experimental results as there is significant mixing enthalpy decrease when considering the Zr_2_(Al_1−x_Bi_x_)C MAX phase with respect to the end-members. This also demonstrates that an intermediate *211* MAX phase can form without the end members forming. If Zr_2_AlC is most probably “simply” unstable or less stable than competing phases (ZrC + Zr_y_Al_z_), Zr_2_BiC experimental non-stability may stem from an increase in Bi covalence strength for high Bi contents, as observed in DFT calculations. The inclusion of Bi is a significant extension of the MAX phase family and this should lead to further investigation of its material properties and technological applications. As an example, Zr_2_(Al_0.42_Bi_0.58_)C may be a possible precursor for Zr_2_C MXene synthesis.

## Methods

### Experimental methods

Zirconium dihydride (ZrH_2_, Alfa Aesar, −325 mesh, >99.7%), bismuth (Alfa Aesar, −325 mesh, >99.5%), aluminium (Alfa Aesar, −325 mesh, >99.5%) and graphite (Sigma-Aldrich, −500 mesh, >99.9%) were used as reactants. To limit as much as reasonably possible oxygen impurities, the reactants were kept and weighed in Nylon milling jars filled with 10 mm ZrO_2_ balls in an Argon glove box. The jars were sealed and placed for 30 mins at 360 rpm in a rotary mill (Nanjing University Instrument Plant). The mixed powders were then transferred in the glove box, sealed in plastic bottles and kept in these bottles up until synthesis reaction. As is the custom for most MAX phase syntheses, stoichiometries were experimentally adjusted to 2/1.05/0.95 for Zr, Al + Bi and C, respectively. This is in order to compensate for the usual partial sublimation of the A element(s) and the possible partial uptake of carbon from the employed graphite crucibles and dies. Reactions were done by pressureless heating of the powders. Minutes before the thermal treatment, the sealed Ar-filled bottles were opened and poured in graphite crucibles (custom-made by Almath Crucibles Ltd., UK) which were then placed in a furnace under argon (FCT Systeme HP W/25/1, Rauenstein, Germany). Three different synthesis temperatures were tested: 1450 °C (1 h plateau), 1300 °C (10 h) and at 1150 °C (10 h). Heating and cooling rates were set at ~20 °C.min^−1^. The obtained compounds were then analysed by XRD using a Bruker D2 Phaser SSD160 (Karlsruhe, Germany). Routine analyses consisted of 6 to 105^o^ 2θ scans with a 0.03^o^ 2θ step and 0.4 sec.step^−1^. Crystalline phase determination was done with the help of Xpert High Score Plus software using ICDD (International Centre for Diffraction Data) database. As Zr_2_BiC and Zr_2_AlC are obviously not reported in the database Zr_2_PbC and Zr_2_SC were used as references for tentative matching. Similarly, the presence of higher order MAX phase (*n* = 2 or 3) was ruled out by comparison with simulated patterns using Crystal Maker and Crystal Diffract software. Refinement of unit cell parameters was done by full-pattern matching (Le Bail function) using the Fullprof Suite program^38^. The powder obtained after 1300 °C synthesis of Zr_2_(Al_0.5_Bi_0.5_)C was manually milled in an agate mortar and underwent a <250 μm sieving to be used for SPS sintering. This was done at Nanoforce lab., Queen Mary University of London, using a HP D/25/1 FCT System equipment and 30 mm graphite cylindrical mold jacketed with graphite paper. Sintering was done at 1300 °C during 10 minutes, this temperature being reached in about 10 minutes. SEM imaging and EDX analyses were performed at 20 keV using a JEOL SEM 6400 equipped with an INCA detector (ultra-thin polymer window, Oxford Instruments, Oxford, UK).

### Computational methods

DFT calculations were performed on the Zr_2_(Al_1−x_Bi_x_)C system using the VASP program. The GGA-PBE exchange correlation was used with pseudopotentials available in the library distributed with the VASP program^39^ (using the highest valence electron version available). Calculations were carried out in a 4 × 4 × 1 supercell (unit cell displayed in Fig. 1), containing 32 formula units (128 atomic sites). A 4 × 4 × 4 γ-centered k-point grid was utilized for all calculations providing a spacing of 0.02 Å^−1^. A Methfessel-Paxton smearing method was used for all calculations with a smearing width of 0.15 eV. All calculations were performed under constant pressure, allowing all atomic positions, lattice parameters and angles to fully relax. The DOS of each system was modelled from the fully geometry optimized state.

Complete random mixing on the A sublattice was investigated by randomly populating Bi and Al species over the 32 available lattice sites. Seven different deviations in stoichiometry were investigated, namely: x = 0.25, 0.33, 0.41, 0.50, 0.59, 0.66 and 0.75 (in Zr_2_(Al_1−x_Bi_x_)C). Ten random supercells of each stoichiometry were generated and relaxed using the method described. The standard deviation in energy was less than 0.02 eV/per Zr_2_(Al_1−x_Bi_x_)C unit.

Formation enthalpies *H*_*form*_ were calculated from the constituent elements (i.e. Zr metal, Al metal, Bi metal and C). The mixing enthalpy *H*_*mix*_ was subsequently calculated from these values, to highlight any preferred stoichiometry that forms as an intermediate composition (negative deviations of mixing enthalpies highlight a preferential system stoichiometry).


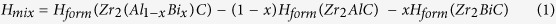


## Additional Information

**How to cite this article**: Horlait, D. *et al.* Synthesis and DFT investigation of new bismuth-containing MAX phases. *Sci. Rep.* **6**, 18829; doi: 10.1038/srep18829 (2016).

## Figures and Tables

**Table 1:** Summary of synthesis results.

Targeted compound	Reaction temperature	Observed phases
Zr_2_AlC	1450 °C	ZrC + ZrAl_2_
Zr_2_(Al_0.50_Bi_0.50_)C		ZrC + ZrAl_2_ + Unknown
Zr_2_BiC		ZrC + Bi + Unknown
Zr_2_AlC	1300 °C	ZrC + ZrAl_2_
Zr_2_(Al_0.50_Bi_0.50_)C		**Zr**_**2**_**(Al**_**0.42**_**Bi**_**0.58**_**)C** + ZrC + ZrAl_2_ + Unknown
Zr_2_BiC		ZrC + Bi + Unknown
Zr_2_AlC	1150 °C	ZrC + ZrAl_3_
Zr_2_(Al_0.50_Bi_0.50_)C		**Zr**_**2**_**(Al**_**0.42**_**Bi**_**0.58**_**)C** + ZrC + Zr_5_Al_4_
Zr_2_(Al_0.41_Bi_0.59_)C		**Zr**_**2**_**(Al**_**0.42**_**Bi**_**0.58**_**)C** + ZrC + Zr_5_Al_4_
Zr_2_BiC		ZrC + Bi + Unknown
